# A cohort study of the prognostic and treatment predictive value of SATB2 expression in colorectal cancer

**DOI:** 10.1038/bjc.2012.34

**Published:** 2012-02-14

**Authors:** J Eberhard, A Gaber, S Wangefjord, B Nodin, M Uhlén, K Ericson Lindquist, K Jirström

**Affiliations:** 1 Department of Clinical Sciences, Division of Oncology, Lund University, Skåne University Hospital, 221 85 Lund, Sweden; 2 Department of Clinical Sciences, Division of Pathology, Lund University, Skåne University Hospital, SE-221 85 Lund, Sweden; 3 Department of Proteomics, AlbaNova University Center, Royal Institute of Technology, 106 91 Stockholm, Sweden

**Keywords:** SATB2, prognosis, treatment prediction, colorectal cancer

## Abstract

**Background:**

Special AT-rich sequence-binding protein 2 (SATB2) is a novel diagnostic marker of colorectal cancer (CRC), and loss of SATB2 has been linked to poor survival from the disease. In this study, we validated the prognostic ability of SATB2 expression in a large, prospective CRC cohort.

**Methods:**

Immunohistochemical SATB2 expression was assessed in 527 incident CRC cases from the Malmö Diet and Cancer Study. Kaplan–Meier analysis and Cox proportional hazards modelling were used to explore the impact of SATB2 expression on cancer-specific survival (CSS) and overall survival (OS).

**Results:**

High SATB2 expression was associated with a prolonged CSS in the full cohort (hazard ratio (HR)=0.61; 95% CI 0.41–0.92) and in colon cancer (HR=0.39; 95% CI 0.20–0.75), remaining significant in multivariable analysis of colon cancer (HR=0.49; 95% CI 0.25–0.96), with similar findings for OS. In curatively resected stage III-IV patients, a significant benefit from adjuvant and/or neoadjuvant therapy was observed for SATB2 high tumours (*P*_interaction_=0.037 for OS) and high SATB2 expression in rectal cancer correlated with an enhanced effect of neoadjuvant therapy (*P*_interaction_=0.033 for OS).

**Conclusion:**

High SATB2 expression is an independent marker of good prognosis in colon cancer and may modulate sensitivity to chemotherapy and radiation.

Colorectal cancer (CRC) is one of the most common forms of human cancer worldwide with approximately 1 million new cases detected every year (Parkin *et al*, 2005). Early detection, adequate surgical excision and optimal adjuvant treatment are of critical importance if a favourable outcome is to be achieved. Currently, tumour stage at diagnosis is the most important prognostic factor in CRC and although many efforts have been made to find molecular markers to identify high-risk disease and to select patients for adjuvant treatment, none have proven good enough for use in clinical routine.

Special AT-rich sequence-binding protein 2 (SATB2), a nuclear matrix-associated transcription factor and epigenetic regulator, was initially identified as a gene involved in osteoblast differentiation and craniofacial patterning in humans (FitzPatrick *et al*, 2003; Dobreva *et al*, 2006). Using the Human Protein Atlas (www.proteinatlas.org) as a tool for biomarker discovery, SATB2 was identified as a highly tissue-type-specific protein being predominantly expressed in glandular cells of the lower gastrointestinal tract and in CRC (Magnusson *et al*, 2011). Immunohistochemical analysis of 1882 tumours from nine independent CRC cohorts revealed that SATB2 was expressed in 85% of all tumours, suggesting the utility of SATB2 as a diagnostic marker for CRC, particularly when used in combination with cytokeratin 20 (CK20) (Magnusson *et al*, 2011). In the same study, the tissue-specific expression of SATB2 was further confirmed by extended analysis of tumours from several other major cancer forms, where SATB2 expression was either completely lacking, for example, in prostate, gastric and pancreatic cancer, or sparsely expressed, for example, in breast, ovarian and lung cancer (Magnusson *et al*, 2011). Apart from being a diagnostic biomarker, the role of SATB2 as a prognostic biomarker in CRC has been implicated in another study, where SATB2 was found to be downregulated at the gene expression level in a metastatic CRC cell line and low immunohistochemical expression of SATB2 was demonstrated to be associated with poor prognosis in an analysis of 146 CRC samples (Wang *et al*, 2009).

Here, we examined the prognostic and treatment predictive value of SATB2 expression in a large number (*n*=527) of CRC cases from a prospective population-based cohort study (Larsson *et al*, 2011; Wangefjord *et al*, 2011). In addition, the association between SATB2 expression and immunohistochemical microsatellite instability (MSI) screening status was explored.

## Materials and Methods

### Study group

Until the end of follow-up 31 December 2008, 626 incident cases of CRC had been registered in the prospective, population-based cohort study Malmö Diet and Cancer Study (MDCS) (Berglund *et al*, 1993). Cases were identified from the Swedish Cancer Registry up until 31 December 2007, and from The Southern Swedish Regional Tumour Registry for the period of 1 January to 31 December 2008. All tumours with available slides or paraffin blocks were histopathologically re-evaluated on haematoxylin and eosin-stained slides. Histopathological, clinical and treatment data were obtained from the clinical and/or pathology records. TNM staging was performed according to the American Joint Committee on Cancer (AJCC). Information on vital status and cause of death was obtained from the Swedish Cause of Death Registry up until 31 December 2009. Follow-up started at date of diagnosis and ended at death, emigration or on 31 December 2009, whichever came first. Median follow-up time was 3.35 years (range 0–17.69) for the full cohort (*n*=626) and 6.05 years (range 1.03–17.69) for patients alive (*n*=344). Patient and tumour characteristics of the cohort have been described in detail previously (Larsson *et al*, 2011; Wangefjord *et al*, 2011). Ethical permissions for the MDCS (Ref. 51/90), and the present study (Ref. 530/2008), were obtained from the Ethics Committee at Lund University.

### Tissue microarray (TMA) construction

Tumours with an insufficient amount of material were excluded, and a total number of 557 (89.0%) tumours were suitable for TMA construction. Areas representative of cancer were marked on haematoxylin- and eosin-stained slides and TMAs were constructed as previously described (Kononen *et al*, 1998). In brief, two 1.0 mm cores were taken from each tumour and mounted in a new recipient block using a semi-automated arraying device (TMArrayer, Pathology Devices, Westminster, MD, USA). As demonstrated previously, there was no selection bias regarding the distribution of clinicopathological characteristics between the TMA cohort and the full cohort (Larsson *et al*, 2011).

### Immunohistochemistry and staining evaluation

For immunohistochemical analysis, 4 *μ*m TMA sections were automatically pre-treated using the PT-link system (DAKO, Glostrup, Denmark) and then stained in an Autostainer Plus (DAKO) with a monoclonal anti-SATB2 antibody (AAb025742, Atlas Antibodies, Stockholm, Sweden) diluted 1 : 100. Non-malignant colonic mucosa served as positive internal control and prostate cancer samples, known to be negative for SATB2 (Magnusson *et al*, 2011), were used as negative controls. The estimated fraction of cells with nuclear SATB2 expression was denoted as 0 (0–1%), 1 (2–25%), 2 (26–50%), 3 (51–75%) and 4 (>75%). Nuclear intensity was denoted as no, weak, moderate or strong, with corresponding scores from 0 to 3, referring to the predominant intensity. A combined nuclear score (NS) was constructed by multiplying fraction and intensity. MSI screening status was evaluated using monoclonal antibodies against MLH1 (Clone ES05, DAKO) diluted 1 : 100, PMS2 (Clone A16–4, 556415, BD Pharmingen, San Diego, CA, USA) diluted 1 : 300, MSH2 (Clone FE11, NA27, Calbiochem, San Diego, CA, USA) diluted 1 : 100, and MSH6 (EPR3945, Epitomics, Burlingame, CA, USA) diluted 1 : 100. Immunohistochemical stainings were evaluated as negative when all tumour cells showed loss of nuclear staining. Surrounding stromal cells and present tumour infiltrating lymphocytes served as internal controls for each biopsy core. A nuclear reaction of tumour cells was assessed as a positive staining. MSI screening status was defined in accordance with previous studies (Dahlin *et al*, 2010), whereby tumour samples lacking nuclear staining of MLH1, PMS2, MSH2 or MSH6 were considered to have a positive MSI screening status. Hereafter, tumours with a positive MSI screening status are referred to as MSI and tumours with negative MSI screening status are referred to as MSS.

The immunohistochemical stainings were evaluated by three independent observers, who were blinded to clinical and outcome data. Scoring differences were discussed in order to reach consensus.

### Statistical analysis

*χ*^2^ and Spearman's correlation (*R*) tests were used to explore the associations between SATB2 expression and relevant clinicopathological and tumour biological characteristics. Classification and regression tree (CRT) analysis was used to decide an optimal cutoff for survival analysis. Kaplan–Meier analysis and log rank test were used to illustrate differences in cancer-specific survival (CSS) and overall survival (OS) according to three categories of SATB2 expression; negative expression (NS=0), intermediate expression (NS 1–9) and high expression (NS>9), the latter corresponding to the optimal prognostic cutoff derived from CRT analysis. Cox regression proportional hazard models were used for estimation of hazard ratios (HRs) for death from CRC or overall causes according to high and low SATB2 expression using the CRT-based cutoff in both uni- and multivariable analysis, adjusted for age, gender, TNM-stage, differentiation grade and vascular invasion. A backward conditional selection method was used for variable selection by the model. The interaction between SATB2 expression and adjuvant and neoadjuvant therapy was explored by a Cox proportional hazards model including a treatment variable and an interaction variable. All tests were two-sided. A *P*-value of 0.05 was considered significant. All statistical analyses were performed using SPSS Statistics version 18 (SPSS Inc., Chicago, IL, USA).

### Remark criteria

A description of the fulfilment of REMARK criteria (McShane *et al*, 2005) for biomarker studies is provided in Supplementary Table 1.

## Results

### Distribution of SATB2 staining and association with clinicopathological characteristics

**Figure 1:**
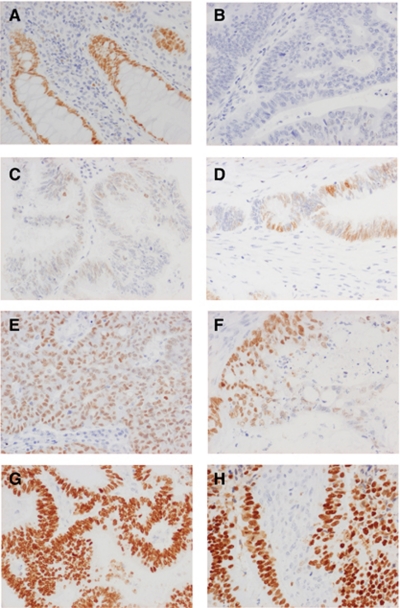
Immunohistochemical images of SATB2 staining in CRC. Images (20 × magnification) representing immunohistochemical expression of SATB2 staining in (**A**) normal colorectal mucosa and CRC, ranging from (**B**) negative through (**C**) weak intensity, (**D** and **E**) moderate intensity in various fractions, (**F**) strong intensity in <75% of tumour cells and (**G** and **H**) strong intensity in >75% of tumour cells.

Following antibody optimisation and staining, SATB2 expression could be evaluated in 527 out of 557 (94.6%) of the tumours represented in the TMA. Examples of immunohistochemical stainings are given in Figure 1. Normal colonic mucosa generally showed moderate to strong SATB2 staining in the majority of cells (Figure 1A). In CRC, SATB2 staining ranged from negative (Figure 1B) through various fractions of weak, moderate and strong staining (Figures 1C–H). The full distribution of SATB2 staining (NS) in all tumours, colon and rectum, is visualised in Supplementary Figures 1A–C. The vast majority of tumours with strong SATB2 intensity had a nuclear fraction of >75% (Figures 1G–H) and only a few had a nuclear fraction of 50–75% (Figure 1F), hence the low number (*n*=4) of tumours denoted as having NS=9 (Supplementary Figure 1A), whereas an NS of 8 (*n*=104) would correspond to moderate staining in >75% of tumour cells (Figure 1E). In the complete evaluated cohort, 152 out of 527 (28.8%) tumours were negative for SATB2. SATB2 expression was lower in colon cancer with 101 out of 321 (31.5%) negative tumours compared with rectal cancer, where 46 out of 193 (23.8%) tumours were SATB2 negative**,** although this difference did not reach statistical significance (*R*=0.081, *P*=0.066). There was no significant difference in SATB2 expression according to neoadjuvant treatment in rectal cancer (data not shown). A total of 13 cases were excluded from the subgroup analyses according to location, 12 (2.3%) cases with multiple synchronous tumours and 1 (0.2%) case with missing information on tumour location.

Next, we examined the relationship between SATB2 expression and established clinicopathological and investigative parameters in the full cohort, colon and rectal cancer, respectively (Table 1). As CRT analysis suggested an optimal cutoff point at NS >9 to determine the impact of SATB2 expression of CSS and OS (Supplementary Figures 1D-E), three categories of SATB2 expression were constructed for comparison of variable distribution: SATB2-negative tumours (NS=0), corresponding to the diagnostic threshold, an intermediate category (NS 1–9) and SATB2 high tumours (NS>9), corresponding to the prognostic threshold derived from CRT analysis. In the full cohort, there was a significant inverse association between SATB2 expression and T stage, N stage, differentiation grade and vascular invasion. All these associations remained significant in colon cancer, whereas in rectal cancer, only the negative association with vascular invasion remained significant (Table 1). There was no significant association between SATB2 expression and age, sex and M stage, neither in the full cohort nor in subgroups according to location. MSI status could be assessed in 515 (92.5%) cases, of which 77 (15.0%) were denoted as MSI, and 438 (85.0%) as MSS, which is in line with previous studies (Dahlin *et al*, 2010). Examples of immunohistochemical stainings of MMR proteins are given in Supplementary Figure 2. The vast majority (68 out of 77; 88.3%) of MSI cases were located in the colon. A highly significant inverse correlation was seen between SATB2 expression and MSI tumours in the full cohort and in colon cancer (Table 1).

### Association between SATB2 expression and survival

**Figure 2:**
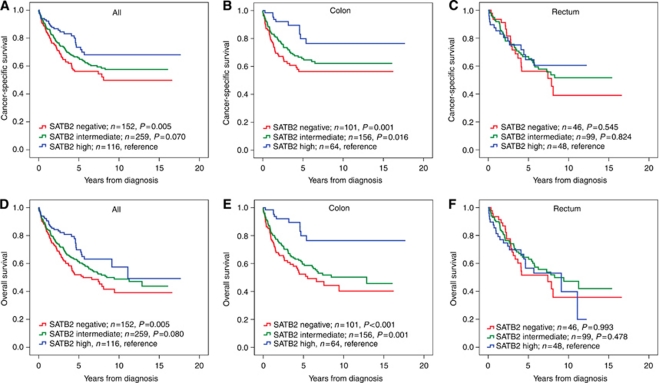
Kaplan–Meier estimates of CRC-specific survival and OS in all patients, and patients with cancer in the colon and rectum. Kaplan–Meier analysis of CRC-specific and OS in strata of negative, intermediate and high SATB2 expression in (**A** and **D**) all patients, (**B** and **E**) colon cancer and (**C** and **F**) rectal cancer. The categories of staining were determined according to the NS, for example, multiplier of fraction and intensity, whereby negative expression=NS 0, intermediate expression=NS 1–9 and strong expression=NS >9.

Next, the impact of negative, intermediate and high SATB2 expression on CSS and OS was determined. In the full cohort, Kaplan–Meier analysis demonstrated a stepwise impairment of CSS and OS with decreasing SATB2 expression (Figures 2A and D). In colon cancer, the beneficial prognostic impact of high SATB2 expression (NS>9) was even more accentuated (Figures 2B and E), whereas no prognostic value was seen for SATB2 expression in rectal cancer (Figure 2C and F). The associations between SATB2 expression and survival were confirmed in univariable Cox regression analysis using the CRT-defined cutoff to define categories of high and low expression (Table 2). In multivariable analysis, SATB2 remained an independent prognostic factor for both CSS (HR=0.49, 95% CI 0.25–0.96, *P*=0.039) and OS (HR=0. 37, 95% CI 0.19–0.71, *P*=0.003) in patients with colon cancer, but not in the full cohort (Table 2). SATB2 was not prognostic in MSI tumours (data not shown). We confirmed that exclusion of cases with missing information in any of the covariates or inclusion of MSI status in the multivariable analysis did not substantially alter the results (data not shown). The prognostic value of SATB2 expression did not differ according to gender (data not shown). Univariable Cox regression analysis using a dichotomised variable of negative (NS=0) *vs* any (NS>0) SATB2 expression showed that the latter was significantly associated with a prolonged CSS and OS in the full cohort and colon, but not rectal, cancer. These associations were, however, lost in multivariable analysis (data not shown).

The distribution of clinicopathological characteristics in patients with colon and rectal cancer in the evaluated cohort are shown in Supplementary Table 2. Patients with rectal cancer were significantly younger at diagnosis (*P*<0.001), had tumours with lower T stage (*P*=0.003) irrespective of radiotherapy (RT) (data not shown), lower differentiation grade (*P*=0.026), and a lower frequency of acute surgery (*P*=0.001). None of the patients with colon cancer had received neoadjuvant RT or chemotherapy and there was no significant difference regarding adjuvant or palliative treatment between patients with colon and rectal cancer. The distribution of clinicopathological characteristics did not differ between the full cohort (*n*=626) and the evaluated cohort (*n*=527) (data not shown).

### Associations between SATB2 expression and response to adjuvant and neoadjuvant therapy

SATB2 was not prognostic in patients with non-metastatic (stage I-II) disease, neither in the full cohort (*n*=263) nor in subgroups according to location (data not shown). However, in patients with stage III-IV disease (*n*=236), high SATB2 expression was associated with a significantly prolonged CSS (HR=0.55, 95% CI 0.33–0.93, *P*=0.025) and OS (HR=0.60, 95% CI 0.37–0.98, *P*=0.041) and in multivariable analysis, this association remained significant for CSS (HR=0.52, 95% CI 0.28–0.95, *P*=0.034) and borderline significant for OS (HR=0.60, 95% CI 0.35–1.03, *P*=0.060) (Table 3). Subgroup analysis according to location in patients with stage III-IV disease revealed a trend, however non-significant, towards an improved CSS and OS for SATB2 high tumours in colon cancer, which was not evident in rectal cancer (data not shown). In curatively treated stage III-IV patients (*n*=134) evaluated for SATB2 expression, including 13 patients with M1 disease, the prognostic value of SATB2 expression was more evident in patients that had received adjuvant chemotherapy than in the untreated group, although no significant interaction could be demonstrated (Table 3). When both adjuvant and/or neoadjuvant therapy (RT and/or chemotherapy) was compared with no treatment in curatively treated stage III-IV patients, a significant interaction between SATB2 and treatment was observed for OS (*P*_interaction_=0.037) and borderline significant for CSS (*P*_interaction_=0.064).

**Figure 3:**
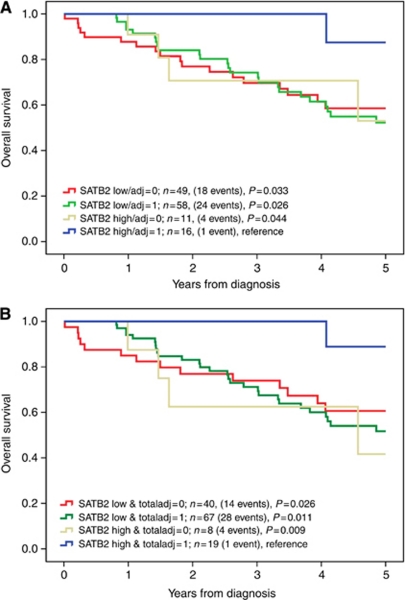
Kaplan–Meier estimates of OS after 5 years according to combinations of SATB2 expression and adjuvant and neoadjuvant treatment. Five-year OS in combined strata according to (**A**) SATB2 expression and adjuvant chemotherapy and (**B**) SATB2 expression and adjuvant chemotherapy and/or neoadjuvant therapy (total adjuvant) in 134 curatively treated patients with stage III-IV disease. SATB2 expression was denoted as low (NS⩽9) or high (NS>9). Log rank *P*-values correspond to pairwise comparisons of SATB2 high and treated tumours with the other strata, respectively.

Kaplan–Meier analysis and the log rank test were also applied to compare long-term and 5-year OS in strata according to combinations of SATB2 expression and adjuvant chemotherapy as well as adjuvant chemotherapy and/or neoadjuvant therapy (mainly RT) in curatively treated patients with stage III-IV disease (Figure 3). This revealed a significantly improved 5-year OS compared with all other strata for patients with SATB2 high tumours receiving adjuvant chemotherapy (Figure 3A) and/or neoadjuvant therapy (Figure 3B). The association between SATB2 expression and treatment benefit was similar for 5-fluorouracil (5-FU) alone or in combination with oxaliplatin (data not shown).

Given the lack of prognostic significance of SATB2 expression in rectal cancer, we also examined whether SATB2 expression might affect response to neoadjuvant RT and/or chemotherapy in patients with rectal cancer. Data on neoadjuvant treatment and SATB2 expression was available for 172 cases, of whom 125 (64.8%) had not received neoadjuvant RT, 44 (25.6%) had received RT, 2 (1.2%) had received radiochemotherapy and 1 (0.6%) patient had received chemotherapy alone (Supplementary Table 2). This revealed a significant interaction between SATB2 expression and neoadjuvant treatment (*P*_interaction_=0.033) in relation to OS (Table 3), also when RT only was included in the treatment variable (*P*_interaction_=0.046). These findings imply that SATB2 expression might also positively affect response to neoadjuvant RT in patients with rectal cancer.

When tumours were stratified into SATB2 negative (NS=0) and SATB2 positive (NS>0), the impact on survival did not differ in strata according to adjuvant and/or neoadjuvant treatment (data not shown).

## Discussion

This study was conducted to examine the prognostic impact of SATB2 expression in incident CRC cases from a large prospective, population-based cohort study. SATB2 has previously been described as a promising novel diagnostic marker for CRC (Magnusson *et al*, 2011) and, in a smaller CRC cohort, loss of SATB2 has been linked to poor prognosis (Wang *et al*, 2009). We found that high expression of SATB2 was an independent factor of good prognosis in colon but not rectal cancer. Moreover, in curatively treated patients with stage III–IV disease, SATB2 expression was a predictor of response to adjuvant chemotherapy, irrespective of tumour location, and in patients with rectal cancer, a significant interaction between high SATB2 expression and response to neoadjuvant therapy was observed. The findings of an association between SATB2 and an improved response to chemotherapy and radiation therapy are of potential interest, but should be interpreted with caution, as treatment data was not available for all patients in this cohort, hence only allowing for rather small subgroup analyses. On the other hand, since the MDCS started as early as in the mid 90s, when adjuvant chemotherapy was not yet standard of care in Sweden, the comparatively high proportion of patients with stage III tumours not receiving adjuvant treatment is a relative strength in the use of this cohort for biomarker studies. Nevertheless, the putative treatment predictive role of SATB2 should preferably be validated in tumour specimens from randomised, controlled treatment trials and the molecular basis for how SATB2 might modulate the effects of chemotherapy and radiation also remains to be elucidated. To date, the role of SATB2 in chemotherapy response has only been investigated in one study on head and neck squamous cell carcinoma (HNSCC) cells, where SATB2 was demonstrated to promote chemo- and radiation resistance by modulation of ΔNp63 (Chung *et al*, 2010). These findings are in contrast to ours but, notably, in the same study, immunohistochemical detection of SATB2 was reported in more than 50% of human HNSCC tumours, which is not consistent with the antibody-based screening in the Human Protein Atlas, where SATB2 could not be detected in HNSCC using different well-validated antibodies (www.proteinatlas.org). Moreover, the role of SATB2 in transcriptional regulation and as a driver of epigenetic events may well differ between different cancer forms.

The reduced expression of SATB2 in MSI tumours is consistent with studies on other markers of colorectal lineage, for example, CDX2 and CK20 (Lugli *et al*, 2008). Furthermore, in light of the findings from several studies suggesting that 5-FU negatively affects outcome for microsatellite unstable tumours (Barratt *et al*, 2002; Ribic *et al*, 2003; Kim *et al*, 2007), the herein observed association between SATB2 expression and MSI status fits with the improved benefit from adjuvant treatment seen for patients with SATB2 high tumours.

The differential prognostic impact of SATB2 expression in colon and rectal cancer is noteworthy and further underlines the importance of preserving a distinction between the two disease entities, which should also be considered in future validatory studies. In our study, SATB2 expression was found to be higher in rectal cancer compared with colon cancer, although this difference did not reach statistical significance. Additional studies are warranted to clarify whether this finding is coincidental or actually mirrors different tumour biological properties of colon and rectal cancers. As all rectal tumour samples were taken from post-treatment surgical specimens, it could be speculated that SATB2 levels are modified by neoadjuvant RT or chemotherapy. This is however less likely, as SATB2 expression did not differ between treated and untreated tumours.

SATB2 is closely related to SATB1, another member of the SATB family of transcription factors (Kohwi-Shigematsu *et al*, 1997; Yasui *et al*, 2002; Cai *et al*, 2003; Cai *et al*, 2006). Although the role of SATB1 has been more extensively explored in the context of cancer, its impact on prognosis seems to be cancer –type-dependent. In breast cancer, a role for SATB1 as being a master switch towards a metastatic phenotype and a marker of poor prognosis has been demonstrated in a study including immunohistochemical analysis of >1000 human breast cancer specimens (Han *et al*, 2008). In a recent study on rectal cancer (*n*=93), SATB1 expression was found to correlate with a more advanced TNM stage; however, its impact on recurrence or survival was not evaluated (Meng *et al*, 2011). Wang *et al* (2009) also found an inverse association between SATB1 and SATB2 in CRC cells *in vitro*. In lung cancer, a significant loss of SATB1 expression was found in squamous preinvasive lesions and in non-small cell lung cancers compared with matched normal bronchial epithelium, and loss of SATB1 was an independent predictor of poor survival in squamous cell carcinomas (Selinger *et al*, 2010).

The frequency of SATB2-negative tumours reported here is lower than that in the study by Magnusson *et al* (2011), but in that study only ∼10% of the tumours had metastatic disease compared with ∼17% in this cohort, which might in part explain these differences, although SATB2 expression was not found to be significantly associated with M stage, only T and N stage, in this study. A significant association between low SATB2 expression and metastatic CRC was, however, demonstrated in the study by Wang *et al* (2009), where ∼30% of the patients had M1 disease and the frequency of SATB2 low tumours was >50%, but as the proportion of tumours lacking SATB2 expression was not reported, comparisons are difficult to make. Possibly, the lower, although non-significant, frequency of M1 tumours in rectal compared with colon cancers found here could in part explain the observed higher SATB2 expression in rectal cancer. Optimal cutoffs for assessment of the prognostic and treatment predictive value of SATB2 expression will have to be confirmed in future studies. Notably, in this study, although any *vs* negative SATB2 expression was also of prognostic value in univariable analysis, only high expression according to the the CRT-derived cutoff at NS>9, corresponding to the highest expression level, was an independent favourable prognostic as well as treatment predictive factor.

As the Malmö Diet and Cancer Study is a population-based cohort study, a potential selection bias compared with the general population must be taken into consideration (Berglund *et al*, 1993). However, the distribution of clinical stages at diagnosis is in line with the expected, with no favour of less advanced stages. As data on disease recurrence was not available for this study, the impact of SATB2 on recurrence-free survival, not least local recurrence in rectal cancer, should be assessed in future studies, preferably in cohorts where this information has been recorded prospectively.

In conclusion, the findings from this large cohort study demonstrate that high SATB2 expression is an independent factor of good prognosis in colon cancer and imply a putative role for SATB2 in mediating increased sensitivity to chemotherapy and radiation therapy in CRC. The mechanistic basis for these observations should be addressed in future studies.

## Figures and Tables

**Table 1:** Association between SATB2 expression and clinicopathological parameters in all tumours, colon and rectum

	**All tumours**				**Colon**				**Rectum**			
**SATB2 expression *n* (%)**	**Negative 152 (24.3)**	**Intermediate 259 (41.4)**	**High 116 (18.5)**	***P-*value**	**Negative 101 (27.8)**	**Intermediate 156 (43.0)**	**High 64 (17.6)**	***P-*value**	**Negative 46 (19.9)**	**Intermediate 99 (42.9)**	**High 48 (20.8)**	***P-*value**
*Age*												
⩽75	109 (71.7)	165 (63.7)	82 (70.7)	0.683	68 (67.3)	91 (58.3)	41 (64.1)	0.477	38 (82.6)	73 (73.7)	38 (79.2)	0.710
>75	43 (28.3)	94 (36.3)	34 (29.3)		33 (32.7)	65 (41.7)	23 (35.9)		8 (17.4)	26 (26.3)	10 (20.8)	
												
*Gender*												
Female	78 (51.3)	133 (51.4)	66 (59.6)	0.407	56 (55.4)	83 (53.2)	36 (56.2)	0.998	18 (39.1)	49 (49.5)	28 (58.3)	0.063
Male	74 (48.7)	126 (48.6)	50 (43.1)		45 (44.6)	73 (46.8)	28 (43.8)		28 (60.9)	50 (50.5)	20 (41.7)	
												
*T stage*												
1–2	19 (13.0)	62 (25.0)	28 (25.2)	0.002^a^	11 (11.1)	31 (20.3)	14 (21.9)	0.011^b^	8 (19.0)	31 (34.1)	14 (32.6)	0.145
3	96 (65.8)	153 (61.7)	70 (63.1)		64 (64.6)	97 (63.4)	42 (65.6)		29 (69.0)	54 (59.3)	26 (60.5)	
4	31 (21.2)	33 (13.3)	13 (11.7)		24 (24.2)	25 (16.3)	8 (12.5)		5 (11.9)	6 (6.6)	3 (7.0)	
*Missing*	*6*	*11*	*5*		*2*	*3*	*0*		*4*	*8*	*5*	
												
*N stage*												
0	67 (46.9)	147 (61.8)	65 (65.0)	0.002^a^	41 (43.2)	93 (63.3)	38 (64.4)	0.003^a^	24 (54.5)	51 (58.6)	24 (64.9)	0.367
1	42 (29.4)	55 (23.1)	20 (20.0)		28 (29.5)	35 (23.8)	10 (16.9)		13 (29.5)	19 (21.8)	9 (24.3)	
2	34 (23.8)	36 (15.1)	15 (15.0)		26 (27.4)	19 (12.9)	11 (18.6)		7 (15.9)	17 (19.5)	4 (10.8)	
*Missing*	*9*	*21*	*16*		*6*	*9*	*5*		*2*	*12*	*11*	
												
*M stage*												
0	121 (80.7)	207 (80.9)	103 (90.4)	0.058	77 (77.8)	124 (79.5)	56 (88.9)	0.113	41 (89.19	80 (83.3)	43 (91.5)	0.728
1	29 (19.3)	49 (19.1)	11 (9.6)		22 (22.2)	32 (20.5)	7 (11.1)		5 (10.9)	16 (16.7)	4 (8.5)	
*Missing*	*2*	*3*	*2*		*2*	*0*	*1*		*0*			
												
*Differentiation grade*												
Intermediate-high	94 (63.1)	212 (82.5)	98 (86.7)	<0.001^a^	54 (54.5)	120 (77.9)	56 (88.9)	<0.001^a^	35 (77.8)	89 (89.9)	39 (84.8)	0.350
Low	55 (36.9)	45 (17.5)	15 (13.39		45 (45.5)	34 (22.1)	7 (11.1)		10 (22.2)	7 (15.29	7 (15.2)	
*Missing*	*3*	*2*	*3*		*2*	*2*	*1*		*1*	*0*	*2*	
												
*Vascular invasion*												
No	34 (35.8)	76 (51.4)	39 (63.9)	<0.001^a^	20 (32.3)	43 (49.4)	22 (61.1)	0.004^a^	12 (42.9)	30 (52.6)	17 (70.8)	0.047
Yes	61 (64.2)	72 (48.6)	22 (36.1)		42 (67.7)	44 (50.6)	14 (38.9)		16 (57.1)	27 (47.4)	7 (29.2)	
*Missing*	*57*	*111*	*55*		*39*	*69*	*28*		*18*	*42*	*24*	
												
*MSI status*												
MSS	100 (70.4)	220 (87.6)	108 (96.4)	<0.001^a^	58 (60.4)	125 (82.2)	59 (95.2)	<0.001^a^	40 (97.6)	93 (97.9)	46 (100.0)	0.361
MSI	42 (29.6)	31 (12.4)	4 (3.6)		38 (39.6)	27 (17.8)	3 (4.8)		1 (2.4)	2 (2.1)	0 (0.0)	
*Missing*	*10*	*8*	4		*5*	*4*	*2*		*5*	*4*	*2*	

Abbreviations: N1=1–3 positive nodes; N2⩾4 positive nodes; MSI=Microsatellite unstable; MSS=Microsatellite stable; SATB2=special AT-rich sequence-binding protein 2.

Category denoted as negative refers to tumours with SATB2 nuclear score (NS)=0, intermediate to NS 1–9 and strong to NS >9. *P*-values refer to *χ*^2^-test for X × 2 tables. The categories marked as not done and unknown were not included in the analysis.

Overall, 13 cases were excluded from the subgroup analyses according to location, 12 (2.3%) cases with multiple synchronous tumours and 1 (0.2%) case with missing information on tumour location.

a Significant at the 0.01 level.

b Significant at the 0.05 level.

**Table 2:** Cox uni- and multivariable analysis of relative risks of death from colorectal cancer and overall death according to SATB2 expression in all patients, colon and rectal cancer, respectively

	**Colorectal cancer-specific survival**			**Overall survival**		
	**HR (95%CI)**	***P-*value**	***n* (events)**	**HR (95%CI)**	***P*-value**	***n* (events)**
*All*		Univariable			Univariable	
SATB2 low	1.00	0.020	411 (153)	1.00	0.021	411 (186)
SATB2 high	0.61 (0.41–0.92)		116 (27)	0.65 (0.45–0.94)		116 (34)
		Multivariable			Multivariable	
SATB2 low	1.00	0.168	372 (134)	1.00	0.184	372 (161)
SATB2 high	0.72 (0.45–1.15)		100 (21)	0.75 (0.49–1.15)		100 (26)
						
*Colon*		Univariable			Univariable	
SATB2 low	1.00	0.005	257 (91)	1.00	<0.001	257 (116)
SATB2 high	0.39 (0.20–0.75)		64 (10)	0.31 (0.16–0.60)		64 (10)
		Multivariable			Multivariable	
SATB2 low	1.00	0.039	236 (80)	1.00	0.003	236 (101)
SATB2 high	0.49 (0.25–0.96)		59 (10)	0.37 (0.19–0.71)		59 (10)
						
*Rectum*		Univariable			Univariable	
SATB2 low	1.00	0.727	145 (59)	1.00	0.598	145 (67)
SATB2 high	0.90 (0.51–1.59)		48 (15)	1.14 (0.70–1.87)		48 (21)
		Multivariable			Multivariable	
SATB2 low	1.00	0.864	128 (52)	1.00	0.398	128 (58)
SATB2 high	1.07 (0.51–2.24)		37 (9)	1.32 (0.70–2.48)		37 (13)

Abbreviations: CI=confidence interval; HR=hazard ratio; SATB2=special AT-rich sequence-binding protein 2; SATB2 low=nuclear score ⩽9; SATB2 high=nuclear score >9.

Multivariate analysis included adjustment for age (>/⩽75 years), gender, T stage (I-II, III, IV), N stage (0,1,2), M stage (0, 1), differentiation grade (high-intermediate *vs* low) and vascular invasion (absent, present, missing).

**Table 3:** Cox proportional hazards analysis of the impact of SATB2 expression according to adjuvant and/or neoadjuvant treatment in patients with stage III-IV disease and neoadjuvant treatment in patients with rectal cancer

	**Cancer-specific survival**				**Overall survival**			
	**HR (95%CI)**	***P*-value**	***n* (events)**	***P*-value** ^†^	**HR (95%CI)**	***P*-value**	***n* (events)**	***P*-value** ^†^
*All stage III-IV*		Univariable				Univariable		
SATB2 low	1.00		194 (121)		1.00		194 (131)	
SATB2 high	0.55 (0.33–0.93)	0.025	41 (16)		0.60 (0.37–0.98)	0.041	41 (19)	
		Multivariable				Multivariable		
SATB2 low	1.00		176 (105)		1.00		176 (114)	
SATB2 high	0.52 (0.28–0.95)	0.034	37 (12)		0.60 (0.35–1.03)	0.060	37 (15)	
								
*All stage III-IV, curative intent*		Univariable				Univariable		
SATB2 low	1.00		107 (44)		1.00		107 (52)	
SATB2 high	0.45 (0.18–1.14)	0.092	27 (5)		0.54 (0.25–1.20)	0.120	27 (7)	
								
*Stage III-IV, no adjuvant treatment*								
SATB2 low	1.00		49 (19)		1.00		49 (26)	
SATB2 high	0.79 (0.23–2.69)	0.710	11 (3)		0.95 (0.36–2.48)	0.914	11 (5)	
				0.298				0.175
*Stage III-IV, adjuvant treatment*								
SATB2 low	1.00		58 (25)		1.00		58 (26)	
SATB2 high	0.28 (0.65–1.17)	0.080	16 (2)		0.27 (0.66–1.15)	0.076	16 (2)	
								
*Stage III-IV, no neoadjuvant or adjuvant treatment*								
SATB2 low	1.00		40 (13)		1.00		40 (20)	
SATB2 high	1.30 (0.37–4.59)	0.680	8 (3)		1.36 (0.51–3.66)	0.540	8 (5)	
				0.064				0.037
*Stage III-IV, neoadjuvant and/or adjuvant treatment*								
SATB2 low	1.00		67 (31)		1.00		67 (32)	
SATB2 high	0.21 (0.05–0.90)	0.035	19 (2)		0.21 (0.05–0.88)	0.034	19 (2)	
								
*Rectum, stage I-IV, no neoadjuvant therapy*		Univariable				Univariable		
SATB2 low	1.00		94 (32)		1.00		94 (37)	
SATB2 high	1.20 (0.59–2.44)	0.615	31 (10)		1.59 (0.87–2.91)	0.130	31 (15)	
				0.093				0.033
								
*Rectum, stage I-IV, neoadjuvant therapy*								
SATB2 low	1.00		34 (16)		1.00		34 (17)	
SATB2 high	0.31 (0.07–1.36)	0.120	13 (2)		0.29 (0.07–1.27)	0.101	13 (2)	

Abbreviations: CI=confidence interval; HR=hazard ratio; SATB2=special AT-rich sequence-binding protein 2; SATB2 low=nuclear score ⩽9; SATB2 high=nuclear score >9.

*P*-value from multivariable analysis adjusted for T Stage (1–2 *vs* 3 and 4), N stage (0 *vs* 1 and 2), M Stage (0 *vs* 1), age (>/⩽75 years), differentiation grade (high-intermediate *vs* low) and vascular invasion (absent, present, missing). ^†^*P*-value for term of interaction by Cox multivariate analysis including treatment, the binary covariate SATB2 expression, and a term of interaction.
