# Supplementary figures and images for: A cohort study of the prognostic and treatment predictive value of SATB2 expression in colorectal cancer
Source: Br J Cancer. 2012 Feb 14;106(5):931–8. doi: 10.1038/bjc.2012.34 (PMC3305956; doi:10.1038/bjc.2012.34)

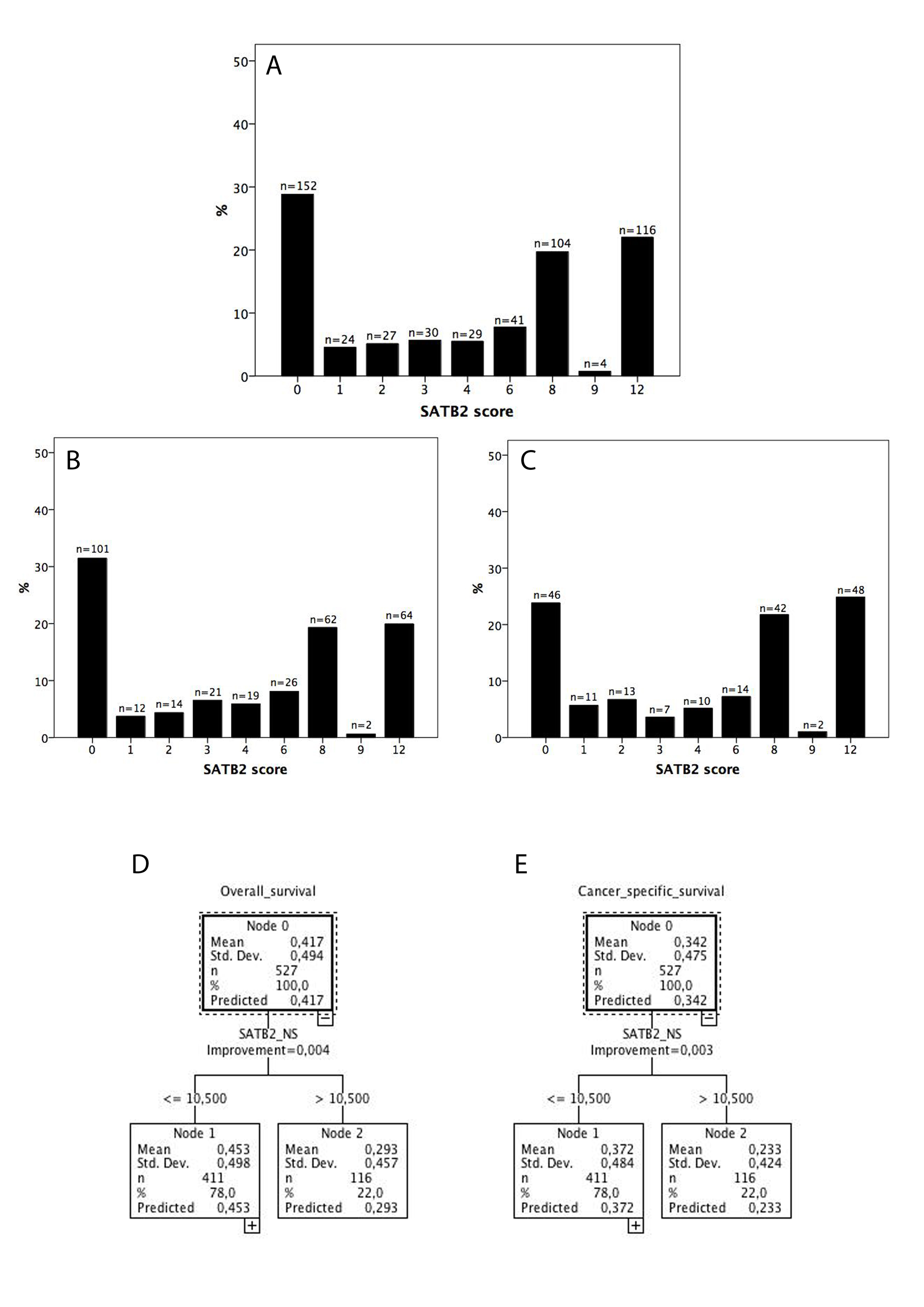

Supplement: Supplementary Figure 1 [file bjc201234x1.tif]

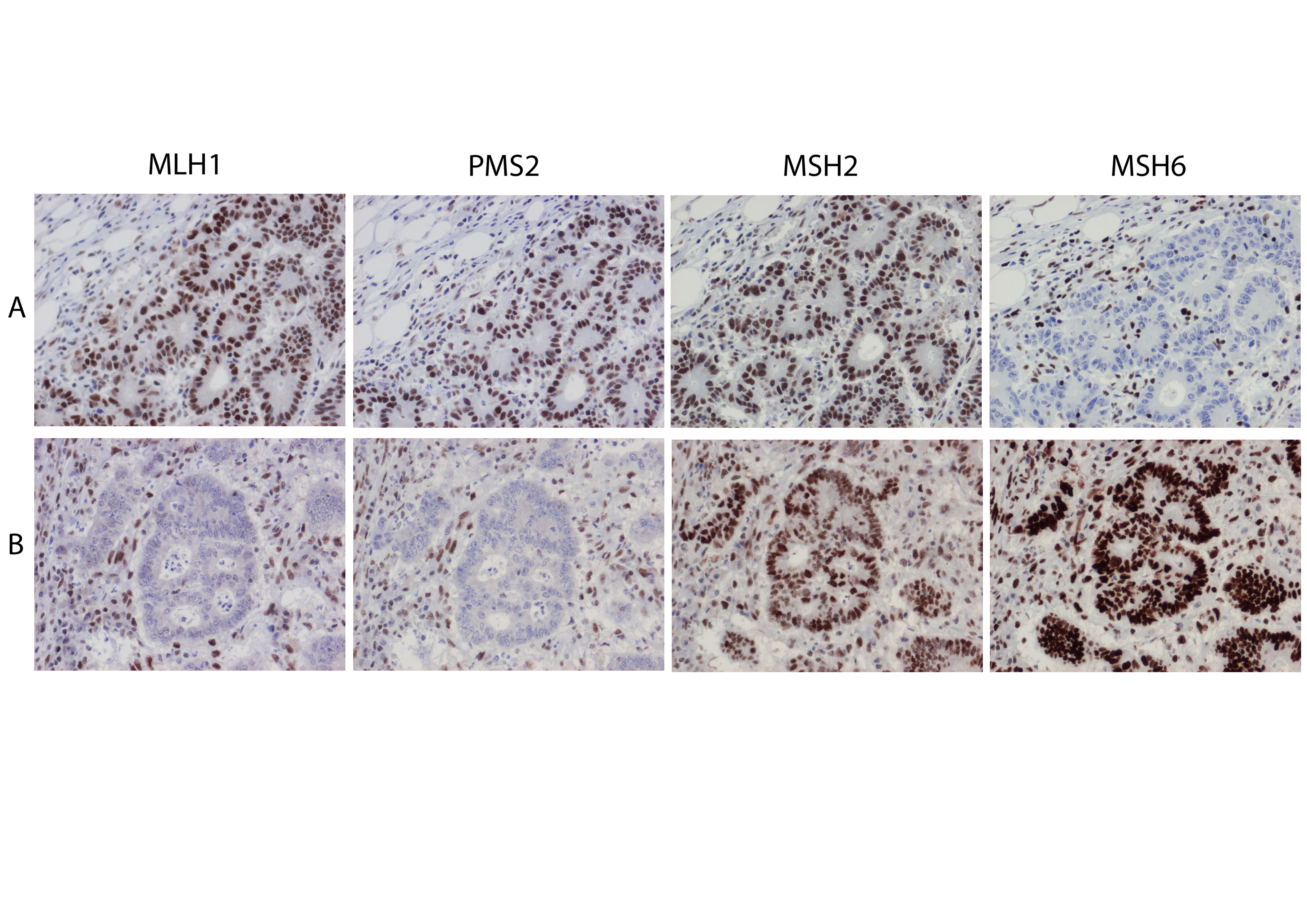

Supplement: Supplementary Figure 2 [file bjc201234x2.tif]
