# Supplementary material for: A cohort study of the prognostic and treatment predictive value of SATB2 expression in colorectal cancer
Source: Br J Cancer. 2012 Feb 14;106(5):931–8. doi: 10.1038/bjc.2012.34 (PMC3305956; doi:10.1038/bjc.2012.34)
Supplement: Supplementary Table 1 [file bjc201234x3.doc]

| **Reporting recommendations for REMARK** | **How criteria are fulfilled** |
| --- | --- |
| **Introduction** |  |
| 1. State the marker examined, the study objectives, and any pre-specified hypotheses | The hypothesis and study objectives are stated on pp 3-4 |
| **Materials and Methods** |  |
| *Patients* |  |
| 1. Describe the characteristics of study patients, including their source and inclusion and exclusion criteria. | The characteristics of the study patients is given on 5, with references. Distribution of clinicopathological parameters in colon and rectal cancer patients is given in Suppl Table 2. This is a population based, prospective cohort study. Details of the study population are described on p 5. |
| 2.Describe treatments received and how chosen | Not applicable |
| *Specimen Characteristics* |  |
| 1. Describe type of biological material used and methods of preservation and storage | The biomarker study is performed on archival paraffin-embedded tumour material, from which suitable specimens were assembled in tissue microarrays all stored and handled in room-temperature. Described on p 5 |
| 2. Specify the assay used and provide (or reference) a detailed protocol, including specific reagents or kits used, quality control procedures, reproducibility assessments, quantitation methods, and scoring and reporting protocols. Specify whether and how assays were performed blinded to the study endpoint. | Details of assays and protocols used are given on pp 6-7.  Assays were performed blinded to the study endpoint. |
| *Study Design* |  |
| 1. State the method of case, selection including whether prospective or retrospective and whether stratification or matching was used. Specify the time period from which cases were taken, the end of the follow-up period, and the median follow-up time. | This is a population based, prospective cohort study. Details of case selection and the time period are given on p 5. |
| 2. Precisely define all clinical endpoints examined. | Details of endpoints are given on p 7. |
| 3. List of all candidate variables initially examined for inclusion in models. | Described on p 7. |
| 4. Give rationale for sample size; if the study was designed to detect a specified effect size, give the target power and effect size. | Not applicable since this study involved incident colorectal cancer cases in a prospective, population-based cohort study. |
| *Statistical analysis methods* |  |
| 5. Specify all statistical methods, including details of any variable selection procedures and other model-building issues, how model assumptions were verified, and how missing data were handled. | Specified on p 7. |
| 6. Clarify how marker values were handled in the analysis. | Clarified on pp 6-7. |
| **Results** |  |
| *Data* |  |
| 1. Describe the flow of patients through the study, including the number of patients included in each stage of the analysis and reasons for drop out. Specifically, report the number of patients and the number of events. | This was not a staged analysis. The evaluated cohort is described on pp 8-12. |
| 2. Report distributions of basic demographic characteristics, standard prognostics variables | Table 1 shows correlations between the investigated marker and standard prognostic variables. Standard prognostic indicators are also included in the multivariate analyses in Table 2 and 3. |
