# Supplementary material for: A cohort study of the prognostic and treatment predictive value of SATB2 expression in colorectal cancer
Source: Br J Cancer. 2012 Feb 14;106(5):931–8. doi: 10.1038/bjc.2012.34 (PMC3305956; doi:10.1038/bjc.2012.34)
Supplement: Supplementary Table 2 [file bjc201234x4.doc]

**Supplementary Table 2**

| **n (% for columns)** | **Colon**  n=321 (62.5) | **Rectum**  n=193 (37.5) | **p-value** |
| --- | --- | --- | --- |
| **Age**  <=75  >75 | 200 (62.3)  121 (37.7%) | 149 (77.2)  44 (22.8) | <0.001 |
| **Sex**  Female  Male | 175 (54.5)  146 (45.5) | 95 (49.2)  98 (50.8) | 0.245 |
| **T Stage**  1  2  3  4  *Unknown* | 30 (9.5)  26 (8.2)  203 (64.2)  57 (18.0)  5 | 16 (9.1)  37 (21.0)  109 (61.9)  14 (8.0)  17 | 0.003 |
| **N stage**  0  1  2  *Unknown* | 172 (57.1)  73 (24.3)  56 (18.6)  20 | 99 (58.9)  41 (24.4)  28 (16.7)  25 | 0.617 |
| **M Stage**  0  1  *Unknown* | 257 (80.8)  61 (19.2)  3 | 164 (86.8)  25 (13.2)  4 | 0.084 |
| **Differentiation grade**  High  Intermediate  Low  *Unknown* | 23 (7.3)  207 (65.5)  86 (27.2)  5 | 9 (4.7)  154 (81.1)  27 (14.2)  3 | 0.026 |
| **Vascular invasion**  No  Yes  *Unknown* | 85 (45.9)  100 (54.1)  136 | 59 (54.1)  50 (45.9)  84 | 0.799 |
| **MSI status**  MSS  MSI  *Unknown* | 245(78.3)  68(21.7)  8 | 185(98.4)  3(1.6)  5 | <0.001 |
| **Surgery**  Acute  Elective  *Unknown* | 39 (13.0)  260 (87.0)  22 | 7 (3.7)  182 (96.3)  4 | 0.001 |
| **Neoadjuvant therapy**  None  Short RT  Long RT  Chemotherapy + RT  Chemotherapy  Unknown | 280 (100.0)  0  0  0  0  41 | 125 (72.7)  26 (15.1)  18 (10.5)  1 (1.2)  1 (0.6)  45 (15.0) | 0.000 |
| **Adjuvant therapy**  No  FLV/XELODA  FLOX/XELOX  Other  Curative; M1*  Palliative  Unknown | 168 (60.6)  34 (12.3)  16 (5.8)  3 (1.1)  10 (3.6)  46 (16.6)  44 | 123 (70.7)  15 (8.6)  2 (1.1)  2 (1.1)  3 (1.7)  29 (16.7)  19 | 0.683 |
| **Follow-up (years)**  Mean  Median  Range | 4.7  3.3  0.0-17.7 | 4.9  3.9  0.08-16.6 | 0.220 |
| Dead from CRC  Dead from overall causes | 101 (31.5)  126 (39.3) | 74 (38.3)  88 (45.6) | 0.111  0.158 |
