# Supplementary material for: A cohort study of the prognostic and treatment predictive value of SATB2 expression in colorectal cancer
Source: Br J Cancer. 2012 Feb 14;106(5):931–8. doi: 10.1038/bjc.2012.34 (PMC3305956; doi:10.1038/bjc.2012.34)
Supplement: Supplementary Figure Legends [file bjc201234x5.doc]

# Supplemental Figure 1. Bar charts visualizing the staining distribution of SATB2 in (A) all tumours, (B) colon and (C) rectal cancer. Values on the X-axis refer to the nuclear score (NS), e.g. a multiplier of fraction (0-4) and intensity (0-3) of staining. Percentage values are shown on the Y-axis and actual numbers within the bars. Classification and regression tree analysis analysis determining an optimal prognostic cutoff at >9 was performed for both (D) colorectal cancer specific and (E) overall survival.

**Supplemental Figure 2.** Examples of immunohistochemical staining (20Xmagnification) of indicated MMR proteins, showing loss of MSH6 expression in case A and loss of MLH1 and PMS2 expression in case B.
